# Can supplementation with antioxidants improve cognitive functions in patients with multiple sclerosis? A literature review

**DOI:** 10.1097/MS9.0000000000003124

**Published:** 2025-04-16

**Authors:** Ali Rezaei, Majid Hamidi, Homa Seyedmirzaei, Abdorreza Naser Moghadasi

**Affiliations:** a Multiple Sclerosis Research Center, Neuroscience Institute, Tehran University of Medical Sciences, Tehran, Iran

**Keywords:** antioxidants, cognitive functions, memory, multiple sclerosis, oxidative stress

## Abstract

Multiple sclerosis (MS) is a chronic neuroinflammatory and neurodegenerative disease of the central nervous system (CNS) with a complex and multifactorial pathophysiology. Although these mechanisms are not yet fully elucidated, it is established that oxidative stress (OS) plays a key role in driving neurodegeneration in MS. These pathological mechanisms contribute to a wide range of symptoms, including motor and sensory deficits, as well as cognitive impairment. The impairments in cognitive functions can cause a major burden for these patients and significantly affect their quality of life. For example, memory is one of the most frequently impaired cognitive domains in MS. These deficits often correlate with biomarkers of neurodegeneration and disease progression. Despite the substantial burden of cognitive impairment in MS, no established treatments currently exist to prevent or mitigate cognitive decline in these patients, aside from the disease-modifying treatments. Several clinical trials have investigated the potential of antioxidant supplementation to improve cognitive outcomes in MS patients. However, their findings are often controversial. This review discusses trials evaluating the effects of supplementation with various antioxidants, including *Ginkgo biloba*, melatonin, omega-3 fatty acids, vitamins A, N-acetylcysteine, lipoic acid, xanthophylls, and crocin, on cognitive performance. We discuss the findings of these studies, highlight methodological limitations, and explore the underlying mechanisms by which these compounds may modulate cognition. These mechanisms range from mitigating OS, inflammation, and glutamate-induced neurotoxicity in the CNS to addressing secondary symptoms such as depression and fatigue, which are often linked to cognitive decline. By reviewing the current evidence, this review not only underscores the therapeutic potential and limitations of antioxidant supplementation but also provides guidance for future research to optimize study design and advance our understanding of cognitive preservation strategies in MS.

## Introduction

Multiple Sclerosis (MS) is a chronic autoimmune disorder characterized by demyelination and neurodegeneration in the central nervous system (CNS), resulting in a diverse range of symptoms arising from neurological dysfunction in these patients^[1]^. While it was first believed that neuroinflammation and the resulting demyelination are the sole pathophysiological processes in MS, it was later shown that several mechanisms are involved in varying quantities in different stages of this disease. Glutamatergic neurotoxicity and oxidative stress (OS) are among the two major mechanisms that give rise to neurodegeneration in MS and several other neurodegenerative diseases^[2]^.

**HIGHLIGHTS**

- Clinical studies suggest that melatonin supplementation can improve cognitive function in MS patients, especially through enhanced sleep quality, though further research is needed.
- Clinical studies on GbE supplementation in MS patients have shown mixed results, with some improvement in memory scores; however further research with longer durations and better design is needed to assess its potential therapeutic effects.
- Alpha-linolenic acid (ALA), found in plant-based sources like flaxseed and walnuts, has shown potential benefits in MS, including slower disease progression and fewer relapses, suggesting that plant-based omega-3s may be more effective than fish oil.
- LA supplementation has been shown to reduce disease activity markers (MMP-9, ICAM-1) and improve walking performance in secondary progressive MS, suggesting its role in slowing disease progression, though it does not directly improve cognition.
- Supplementation with NAC has been linked to improvements in cognitive function in MS patients, potentially through increased cerebral blood flow and enhanced cerebral glucose metabolism.
- Evidence supporting the benefit of antioxidative supplementation for cognition in PwMS is controversial, with multiple mechanisms, including controlling oxidative stress, neuroinflammation, mitochondrial function, and enhancing cerebral blood flow, influencing cognitive outcomes.
- The complex and multifactorial nature of MS pathology suggests that combining antioxidants with different mechanisms of action may offer more significant cognitive benefits, though challenges remain in designing long-term studies to demonstrate clear effects.

OS arises from an imbalance between reactive oxygen species (ROS) production and the cellular antioxidant defense mechanisms and plays an important role in both acute and chronic phases of MS pathology. In sites of disease activity in the CNS, the inflammatory state triggers the production of oxygen and nitrogen free radicals, subsequently causing further release of inflammatory cytokines^[3]^. The intricate interplay between OS and neurodegeneration has also provoked interest in antioxidant interventions as potential adjuvant therapy for neurodegenerative disorders^[2,4]^.

Patients with MS (PwMS) face several challenges during the course of disease progression. Cognitive impairment is one of the burdens PwMS may suffer from, significantly impacting their quality of life^[5]^. Attention, memory, and information processing speed are among the most frequently impaired domains of cognition in PwMS^[6]^. There is currently no approved treatment for slowing cognitive decline in MS, aside from the benefits associated with disease-modifying treatments. Due to the well-established role of OS in the pathogenesis of MS, researchers have examined whether supplementation with antioxidants can improve the cognitive impairment observed in PwMS. This hypothesis is further strengthened by findings from animal studies demonstrating a causal relationship between the induction of OS in the brain and decline in cognition^[7,8]^. In a study by Fukui, *et al*, OS was induced in rats by subjecting them to hyperoxia. These rats then showed a significant decline in the memory compared with control subjects. Notably, supplementation with vitamin E demonstrated a protective effect against this OS-induced memory impairment^[9]^.

The present study reviews existing literature on the impact of antioxidant supplementation on cognition in PwMS. Between December 2023 and January 2024, two authors searched in PubMed, Scopus, and Web of Science for clinical trials exploring the effects of supplementation with antioxidants on cognition, especially memory in PwMS. The search strategy included general terms (“antioxid*” and “oxidative”) alongside specific compounds known for their antioxidative properties. These compounds were selected based on their established antioxidative effects or their relevance to cognitive studies identified during the initial search. These compounds were: *Ginkgo biloba* (Gb), ginseng, omega-3 fatty acids, lipoic acid (LA), *N*-Acetylcysteine (NAC), melatonin, xanthophylls (lutein and zeaxanthin), vitamins A, E, and C, coenzyme Q10 (CoQ10), selenium, saffron/crocin, and polyphenols.

Our review primarily focuses on the antioxidants with clinical evidence of cognitive effects (whether null, positive, or negative) in MS. Consequently, not all the antioxidants identified during the search are discussed in detail. The insight is especially useful for laying the foundation for future research in this field, identifying potentially rewarding interventions, and improving efficiency of future studies by employing more suitable designs. Furthermore, we dive into the molecular routes of effects for these antioxidants and explore the preclinical findings surrounding their function in neuroinflammation and neurodegenerative conditions. This is in order to estimate how much potential they have for future research and what measurements in future trials would help with identifying the effects of supplementation.

## Melatonin

Melatonin, often referred to as the “sleep hormone,” is primarily synthesized by the pineal gland in response to darkness. Beyond its role in regulating circadian rhythm, melatonin has also been recognized for its antioxidant, anti-inflammatory, and neuroprotective properties^[10]^. Melatonin has both direct and indirect antioxidative properties by scavenging free radicals and promoting the activity of other antioxidant enzymes. It can also enhance mitochondrial function^[11]^. Melatonin may play a crucial role in preventing neurodegenerative processes observed in MS, as its antioxidant properties can counteract OS^[12]^. Additionally, melatonin has been shown to modulate immune responses and inhibit pro-inflammatory cytokines, potentially attenuating the inflammatory cascade associated with MS progression^[13]^. In a study by Alghamdi et al., melatonin significantly improved memory impairment induced by cuprizone in a mouse model of multiple sclerosis. However, the study did not determine whether this effect was specifically due to melatonin’s antioxidant and anti-inflammatory properties or other mechanisms^[14]^.

Few clinical studies have explored the effects of melatonin supplementation on cognitive function in MS patients. In a study on relapsing-remitting MS (RRMS) patients by Roostaei, *et al*, a daily dose of 3 mg of melatonin resulted in enhanced scores in the cognitive subdomain of the modified fatigue impact scale (MFIS) compared with placebo (*P* = 0.006). However, paced auditory serial addition test (PASAT) scores did not improve in this study (*P* > 0.05)^[15]^. Notably, melatonin might impact cognitive functions by improving sleep quality. In a placebo-controlled study by Jallouli, *et al*, it was revealed that a single dose of 6 mg of melatonin in patients with RRMS leads to improved cognition the following morning, as measured by Montreal cognitive assessment (MoCA) (*P* = 0.008). This improvement was along with enhanced sleep quality in these patients, which can explain the observed effect^[16]^. Therefore, the antioxidative impacts and the resulting cognitive changes of long-term supplementation with melatonin in PwMS need to be further studied.

### Ginkgo biloba (Gb)

Gb is a tree with a history of use in traditional Eastern medicine. EGb 761 is a standardized extract of its dried leaves, which contains phytonutrients, including flavonoids, terpenoids, and organic acids. These compounds have antioxidative and anti-inflammatory properties via different routes of effect, including ROS scavenging, iron chelation, decrease in lipid peroxidation, activation of the Nrf2 pathway, inhibition of NF-kB, and increased glutathione (GSH) production^[17–22]^. Gb extract (GbE) protects mitochondrial DNA against OS and improves mitochondrial membrane potential, resulting in preserved mitochondrial function^[23–26]^.

Studies have shown that GbE might be beneficial in improving cognition and especially memory in patients with Alzheimer’s disease and vascular dementia^[27–29]^. Animal studies imply different mechanisms by which GbE might be applying its effects on cognition.^[19,30]^. GbE upregulates the expression of brain-derived neurotrophic factor (BDNF) in the hippocampus and increases its plasma concentration, enhancing neuroplasticity. This plant also inhibits the platelet-activating factor, leading to reduced platelet aggregation and enhanced cerebral blood flow (CBF)^[20,31]^. The human brain constitutes only 2% of body weight, yet it consumes a substantial proportion of circulating glucose and oxygen^[32]^. Consequently, CBF plays a vital role in the brain’s metabolism and its essential functions, such as cognition^[33]^. The decline in CBF with aging may be correlated with cognitive impairment, as the evidence suggests^[33]^.

GbE promotes Nrf2 activity in the human body. The Nrf2 pathway is a pivotal mechanism of cellular defense against OS in the human body and is responsible for production of many of the major endogenous antioxidants, including glutathione peroxidase (GPx), GSH, and thioredoxin (Trx). The Nrf2 pathway protects against ferroptosis, a form of programmed cell death following a disruption in iron homeostasis. Ferroptosis is associated with neuronal death and may be a contributive factor in the neurodegenerative processes observed in MS^[34,35]^. On the other hand, NF-kB takes part in pro-inflammatory responses following OS, resulting in increased production of pro-inflammatory cytokines. An imbalance in the activity of Nrf2 and NF-kB is related to neurodegenerative diseases (NDDs). Dimethyl fumarate, a drug currently used as a treatment for MS, has a regulatory effect on these pathways, which is similar to the effects of GbE. Although the exact mechanism of the effect of dimethyl fumarate is not yet known, it may be due to Nrf2 suppressing overactive microglia and astrocytes. This evidence highlights a potential therapeutic pathway for GbE in MS pathology^[36–38]^.

GbE also exerts a wide range of effects on various neurotransmitters, from augmenting dopamine levels in the frontal cortex and striatum, to increasing acetylcholine by inhibiting acetylcholinesterase^[39]^. The latter function is similar to donepezil, which is used for the treatment of Alzheimer’s disease^[19,30]^. Furthermore, GbE can reduce glutamate release in the CNS^[40]^. As discussed before, neurotoxicity following abnormal elevations in glutamate release is among the major contributing factors for neurodegeneration in MS^[2,41]^. Memantine, another drug used for the treatment of Alzheimer’s disease, has a similar function through antagonistic activity against glutamate receptor^[42,43]^. These mechanisms imply a therapeutic potential for GbE in PwMS.

We found three trials investigating cognitive performance in PwMS in association with GbE supplementation. A one-arm trial conducted by Noroozian, *et al*, showed an improvement of 14.10 points in the mean score of the Wechsler memory test (*P* = 0.001), following 8 weeks of supplementation with GbE^[44]^. The remaining two studies were conducted by Lovera et al., featuring a randomized placebo-controlled design, and a duration of 12 weeks^[19,45]^. The initial study was a pilot trial in 2007, investigating 39 participants, and suggested improvement in the GbE group in the stroop color and word test (*P* = 0.015), and memory score of the perceived deficits questionnaire (PDQ) (*P* = 0.015) compared with placebo. There were no improvements associated with GbE when compared with placebo in the long delay free recall score of the California Verbal Learning Test (CVLT), Controlled Oral Word Association Test (COWAT), PASAT, Symbol Digit Modalities Test (SDMT), and Useful Field of View Test (UFOV) (*P* ≥ 0.05). The latter study was published in 2012 and included 120 participants. In this study, the placebo group performed better than the GbE group in the stroop test (GbE-placebo *z* score: 0.5, 95% CI: − 0.9 to −0.1). However, the changes were insignificant when adjusted for baseline test scores (*P* ≥ 0.05). The PDQ scores also did not change in the GbE group compared with placebo (*P* ≥ 0.05), and the changes in CVLT, COWAT, and PASAT also remained insignificant (*P* ≥ 0.05). The authors attributed the observed effect in the pilot trial to chance error, an explanation with which we concur. GbE supplementation was safe in all of these three trials. Antioxidative serum markers were not measured in any of them^[19,44,45]^. Based on these findings, we encourage future studies to incorporate a longer duration of supplementation, since a minimum follow-up duration of 24 weeks is recommended to reach adequate sensitivity^[46]^. We also encourage the evaluation of serum markers of OS and disease activity to be employed in future study designs.

## Omega-3 fatty acids

Long-chain omega-3 polyunsaturated fatty acids (PUFAs) and the potential impact they might have on cognition have garnered the attention of many researchers in recent decades^[47]^. Eicosapentaenoic acid (EPA) and docosahexaenoic acid (DHA) are two of the main long-chain omega-3 PUFAs, and they are predominantly found in fish oil. The human body can synthesize both EPA and DHA from a precursor called alpha-linolenic acid (ALA). ALA is a short-chain omega-3, mainly found in dairy products and certain seeds, such as flaxseed and walnuts. Both EPA and DHA exhibit anti-inflammatory and antioxidative properties.

EPA can lower lipid peroxidation in the human body by upregulating the enzymes paraoxonase 1 and 2 (PON1 and PON2). Studies have reported lower plasma PON activity in RRMS patients during relapse compared with remission and also in the remission phase of RRMS compared with healthy individuals^[48,49]^, suggesting it may play a role in MS activity. DHA, being one of the main components of cell and mitochondrial membranes in the CNS, takes on various structural and physiological responsibilities in neurons. It has indirect antioxidative properties by means of regulating Trx and glutathione systems^[50,51]^. ALA also has anti-inflammatory effects and has shown neuroprotective effects in animal studies^[52]^. EPA and DHA both may also improve mitochondrial function^[53–56]^. Overall, these fatty acids have a wide range of functions in the human body.

Supplementation with omega-3 PUFAs in healthy adults improves levels of serum total antioxidant capacity (TAC), GPx, and malondialdehyde (MDA) (a final product of lipid peroxidation)^[57]^. However, with respect to their therapeutic effects on cognition, strong evidence rules out any impact on cognitive decline with up to two years of supplementation in healthy adults. Furthermore, it also failed to reduce the risk of new neurocognitive illness^[47,58]^. Studies in PwMS also do not show any improvements in disease progression following EPA and DHA supplementation, considering relapse rates or EDSS progression. In contrast to EPA and DHA, there is limited data on the effects of ALA on MS, and the evidence is in favor of a beneficial impact on MS activity. In one study (OFAMS study), two years of supplementation with EPA and DHA in RRMS patients was investigated, and no benefits regarding disease progression was found when compared with placebo^[59]^. The data from this study was later used by Bjornevik et al. in another study. Bjornevik, *et al* analyzed serum levels of ALA in participants of the OFAMS study and found that higher serum ALA was significantly associated with better magnetic resonance imaging (MRI) outcomes. Serum ALA was also associated with fewer relapses and slower EDSS progression, although not statistically significant^[59,60]^. Furthermore, data from two large-scale cohorts also suggest ALA to be the only PUFA associated with a lower risk of MS diagnosis^[61,62]^.

Concerning cognitive outcomes of omega-3 supplements in PwMS, findings from two large-scale cross-sectional studies are controversial. Both studies assessed cognitive impairments using self-reported questionnaires and did not involve cognitive tasks^[63,64]^. In a 2023 trial, Saxby et al. randomly assigned RRMS patients to either the Swank or Wahls diet for 24 weeks. Concurrently, both groups received 5 grams of cod liver oil each day, which is a rich source of EPA and DHA. SDMT and PDQ were used for the assessment of cognitive performance, and both showed improvement in both groups at 24 weeks (*P* < 0.05 and ≤ 0.0002 for both groups, respectively). PDQ was also enhanced at 12 weeks (*P* ≤ 0.0002 for both groups). The changes in serum EPA and DHA at 12 weeks were also associated with changes in SDMT scores (*P* < 0.05). However, both groups were supplemented, and a control group for cod liver oil supplementation was not incorporated in the design of this study. Therefore, it does not account for the learning effect in SDMT scores^[65,66]^.

Based on the presented data, we highly encourage future studies to consider plant-based sources of omega-3, which are high in ALA, such as flaxseed or walnut, over fish oil. This recommendation is grounded in the scarcity of data in this regard, despite the encouraging clinical evidence. However, one possible confounding factor in all omega-3 studies would be the daily consumption of dietary seed oils, which are rich sources of omega-6. The ratio of some omega-6 to omega-3 PUFAs in serum is known to impact inflammatory processes. Therefore, the high intake of dietary omega-6 might be overshadowing the interventions with omega-3s^[67-69]^.

## Lipoic acid (LA)

LA, also known as thioctic acid, is an endogenous organosulfur compound with many antioxidative properties. LA can scavenge ROS and chelate metal ions in the human body, and can also regenerate other antioxidants, such as vitamin E. It is the main cofactor for α-ketoglutarate dehydrogenase, a mitochondrial enzyme involved in the Krebs cycle. α-Ketoglutarate dehydrogenase is one of the main sensors of redox status, regulating metabolism in order to control OS. LA can also indirectly regulate Nrf2 and NF-kB pathways^[70,71]^. In rat models of traumatic brain injury, treatment with LA has enhanced levels of Nrf2 in the cerebral cortex, resulting in inhibited neuronal apoptosis and better neurological outcomes^[72]^.

Studies on PwMS have shown a reduction in serum matrix metalloproteinase-9 (MMP-9) and intercellular adhesion molecule-1 (ICAM-1) levels within as little as two weeks of LA supplementation^[73,74]^. Both of these molecules are associated with T-cell migration across the blood-brain barrier, and disease activity in RRMS. A study with a 12-week regimen of LA showed that it increased TAC in RRMS patients (*P* = 004); although levels of superoxide dismutase (SOD), GPx, and MDA did not improve (*P* ≥ 0.05)^[75]^. LA is also reported to be associated with improvement in walking performance in secondary progressive MS (SPMS) patients^[76]^. In a clinical trial examining 2 years of LA supplementation in SPMS patients, Spain et al. reported a 68% reduction in annualized percent change brain volume in patients taking LA compared with a placebo (*P* = 0.002), although SDMT scores did not improve in these patients (*P* = 0.59)^[77]^. These findings imply that LA may not directly impact cognition in PwMS. However, it may indirectly help with preserving cognitive functions by slowing disease progression.

## *N*-acetylcysteine (NAC)

NAC is a pharmaceutical agent used predominantly for acetaminophen toxicity and as a mucolytic agent in respiratory conditions^[78]^. Enhancing the biosynthesis of GSH in the body and modulating inflammation comprise this compound’s antioxidative functions. This function results in reducing key inflammatory markers, such as TNF-α and IL-6, in the human body^[79,80]^. In a study on rats with cuprizone-induced CNS demyelination, Sharouny et al. observed that oral supplementation with NAC provided neuroprotective effects against demyelination, attributed to its antioxidant properties^[81]^.

NAC influences neurotransmission by regulating glutamate levels in the CNS. This mechanism is not only relevant to the pathogenesis of MS but also to cognition^[82,83]^. Evidence suggests that NAC may support cognitive functions. For example, a study by Pandya, *et al* reported significant cognitive improvement in rats following traumatic brain injury when administered a CNS-permeable form of NAC, attributing this effect to reduced OS and preservation of mitochondrial GSH levels^[84]^. Clinical studies have frequently administered NAC in combination with other substances such as the vitamin B group, LA, GB, etc., resulting in improved cognitive function in conditions like Alzheimer’s disease. However, the specific contribution of NAC remains unclear^[85]^.

Several trials have studied NAC supplementation in PwMS. In a study by Khalatbari, *et al*, an 8-week regimen of NAC in PwMS resulted in a decrease in serum MDA levels. Additionally, there was a notable reduction in anxiety test scores^[86]^. Schipper, *et al* observed a trend toward a reduction in the erythrocyte glutathione disulfide (GSSG) to GSH ratio, indicating an improvement in OS in individuals with RRMS after supplementation with NAC. This study used 2.5 grams of NAC twice daily for 36 weeks^[87]^.

NAC has non-antioxidant mechanisms that may support cognitive health in PwMS. In a recent study, two months of NAC supplementation significantly increased CBF in regions involved in attention, memory, and executive function, such as the pons, midbrain, hippocampus, frontal lobe, temporal lobe, and thalamus^[88]^. This enhancement in CBF is promising, as decreased perfusion in these areas is linked to cognitive impairment^[33,89,90]^. In this study, cognitive improvements were observed in the NAC group, although they did not directly correlate with specific regional CBF changes.

Enhancing glucose metabolism in the CNS may be another way NAC supports cognitive functions in MS. A clinical trial in RRMS patients showed that NAC increased cerebral glucose metabolism, particularly in regions tied to cognition, such as the caudate nucleus, inferior frontal gyrus, and temporal gyri^[91-94]^. Improved glucose utilization has been linked to enhanced cognitive functions, especially verbal performance^[95]^. In the hippocampus – a key region for learning and memory – glucose uptake is critical for synaptic plasticity^[96]^. Given that glucose hypometabolism is a hallmark of Alzheimer’s and other age-related cognitive impairments, NAC’s ability to boost glucose metabolism may represent a promising pathway for its cognitive benefits^[97-99]^.

While NAC’s antioxidant effects are well-documented, its ability to influence CBF and glucose metabolism independently suggests additional mechanisms that may contribute to cognitive benefits in MS. Further studies are needed to clarify the specific contributions of NAC’s antioxidant properties in relation to these other pathways.

## Xanthophylls

Lutein and zeaxanthin, members of the xanthophyll family, are two yellow-colored carotenoids. These xanthophylls are strong direct scavengers of ROS, which in conjunction with their ability to absorb harmful ultraviolet and blue radiation in the eye, prevent OS in the retina and protect against macular degeneration^[100]^. Carotenoids are also present in blood and other tissues in amounts that are interrelated. The predominant carotenoid found in the human brain is lutein. Examination of the human brain and retinal tissues in a study by Vishwanathan et al. in 2016 revealed a correlation between the contents of lutein in the macula and the occipital cortex^[101]^. Another study in 2013 also found similar associations between the retinal and cerebral tissue concentrations for both lutein and zeaxanthin in primates^[102]^. Altogether, these findings imply that measures of macular xanthophylls may potentially indicate their cerebral content.

Macular pigment optical density (MPOD) is a non-invasive measure of macular lutein and zeaxanthin, which makes it convenient for human studies to measure concentrations of these pigments in the macula. Therefore, several studies have been conducted on xanthophylls, employing MPOD as a measurement for their levels in the human body. Evidence shows a positive connection between MPOD and cognition in the elderly.^[101,103]^. Supplementation with lutein and zeaxanthin is also shown to improve MPOD^[104]^. Thus, it suggests that supplementation with these pigments may aid in preserving cognition in the elderly. Moreover, studies in PwMS show a decreased MPOD compared with healthy controls. In a recent study, Cerna, *et al* measured MPOD in PwMS and evaluated their attentional inhibition using the Eriksen flanker task coupled with electroencephalography (EEG). The team found that patients with higher MPOD had better attention control and processing speed (*P* < 0.05)^[105,106]^. These findings denote a potential therapeutic role for xanthophylls concerning cognitive deficits associated with MS, laying the foundation for trials to further investigate this matter.

We found only one study assessing the cognitive effects of supplementation with xanthophylls. In a clinical trial by Martell, *et al*, RRMS patients were administered a four-month supplementation of lutein with a daily dose of 20 mg, and measured indices of lutein content along with a battery of cognitive tasks, including the Eriksen flanker task plus EEG, SDMT, and a spatial reconstruction task. Supplementation with lutein resulted in enhanced skin carotenoids, serum lutein, and MPOD compared with placebo (*P* < 0.01 for all three). This study did not identify a significant increase in measures of cognition in the lutein group (*P* > 0.05). However, it found a correlation between changes in MPOD and accuracy in Eriksen flanker task (*P* = 0.03) as well as spatial memory (*P* = 0.02). This study lacks a measure of cognition that is independent of vision^[107]^. Future studies are encouraged to try longer periods of intervention and employ non-visual cognitive tests.

## Saffron

Saffron (*Crocus sativus*) is another plant with a history of use in some systems of traditional medicine. It contains various compounds such as safranal and crocin that possess antioxidative and anti-inflammatory properties. Saffron extract has demonstrated efficacy in mitigating OS and enhancing learning and memory in animal models of demyelination induced by ethidium bromide. The observed changes in cognition were alongside improvements in TAC, SOD, GPx, and MDA^[108,109]^. Crocin, a major component of saffron extract, is a potent ROS scavenger and also exerts inhibitory effects on NF-kB. In a study on cuprizone-induced animal models of demyelination in the brain, supplementing with crocin significantly resulted in improved levels of MDA, SOD, GPx, and TAC, along with enhanced reflexive motor behaviors, implying a potential therapeutic effect on demyelinating processes^[110]^.

We found two studies investigating the cognitive effects of saffron, safranal, or crocin on cognition in MS. A 2019 congress abstract by Doosti, *et al* investigated cognitive effects of 1 year of supplementation with saffron on RRMS patients. This study was a clinical trial, employing a randomized placebo-controlled and triple-blind design. Using a fairly extensive neuropsychological battery, the authors reported a notable improvement in COWAT (*P* = 0.006) and North American Adult Reading Test (NAART) (*P* = 0.015) in the saffron group compared with placebo^[111]^. Rezaeimanesh, *et al* have also supplemented PwMS with crocin-selenium nanoparticles for 12 weeks. The crocin-selenium group had insignificantly higher scores in CVLT-II and SDMT tests. Based on the findings of the study by Doosti et al.^[111]^, a longer follow-up might have shown statistically significant results. However, TAC was improved in the crocin-selenium group compared with placebo (*P* = 0.01). It is necessary to note that selenium itself has antioxidative effects, therefore this enhancement in TAC cannot be attributed to crocin alone^[112]^. These findings underscore saffron’s potential therapeutic role in the cognition of PwMS, especially within verbal domains. However, no full-text article was yet available from the congress abstract by Doosti et al. during the time of our search. Therefore, the methodology of their study is not available in detail, and the strength of their design remains in question. Future studies are encouraged to explore this matter and check whether these results are repeatable.

## Vitamin A

Vitamin A is a general term for various fat-soluble agents, such as retinol, retinyl palmitate, and beta-carotene, found in animal sources, fruits, and vegetables^[113]^. Mean vitamin A levels are reported to be lower in PwMS than in controls but with borderline significance (*P* = 0.05)^[114]^. According to evidence in experimental autoimmune encephalomyelitis (EAE) animal models, vitamin A inhibits T-helper 17 cells^[115]^, and a lack of T-helper 17’s main cytokine, interleukin-17A (IL-17A), enhances cognitive deficits in mice^[116]^. Treatment with all-trans retinoic acid (a derivative of vitamin A) can reduce the expression of pro-inflammatory cytokine genes in patients with RRMS^[117]^. These properties have attracted researchers to evaluate the therapeutic effects of vitamin A on MS^[118]^.

We identified three studies investigating the effects of vitamin A on cognition in PwMS. In a controlled randomized clinical trial by Bitarafan, *et al*, people with RRMS underwent 25 000IU/day retinyl palmitate for six months, followed by 10 000IU/d retinyl palmitate for another six months. The treatment group showed improved scores in the cognitive subdomain of MFIS (*P* = 0.02)^[119]^ compared with placebo. In another study by Bitarafan, *et al* with the same dose and duration, PASAT scores improved significantly in RRMS patients supplemented with retinyl palmitate compared with placebo (*P* = 0.03)^[120]^. The third study was a cohort by Ruschil et al., that had only 3 cases of progressive MS under treatment with all-trans retinoic acid^[118]^. Their cognition was tested with PASAT and compared with 50 controls over a minimum follow-up duration of 300 days, but no significant changes were found. The low sample size in this study, however, implies a low power and a high chance of type II errors. Therefore, the results of the studies by Bitarafan, *et al* are more robust. In the studies we reviewed, no serious adverse effects were reported for vitamin A, whether administered as retinyl palmitate or all-trans retinoic acid. Future studies can focus on evaluating the repeatability of these findings.

## Vitamin E

Vitamin E, particularly its active form alpha-tocopherol (α-Toc), has potent antioxidant properties that help reduce OS by scavenging ROS, lowering lipid peroxidation, and maintaining cell membrane integrity^[121,122]^. Studies demonstrate that α-Toc can improve cognitive function in various neurodegenerative and cognitive impairment models by reducing inflammation and restoring oxidative balance. For example, in animal models of Alzheimer’s and traumatic brain injury, α-Toc reduced OS markers and neuroinflammatory cytokines^[123]^, while also enhancing long-term potentiation (LTP)^[124]^, a key process in learning and memory^[125,126]^.

In PwMS, vitamin E supplementation has been shown to reduce lipid peroxidation, though no clinical trials directly link it to cognitive outcomes in PwMS^[127]^. However, preclinical studies suggest Vitamin E may aid remyelination and protect against OS-related damage to SNARE proteins, which are essential for neurotransmission and myelination^[128,129]^. We found no clinical evidence of Vitamin E toxicity in PwMS. However, studies using tocopherols and tocotrienols in EAE animal models have reported an associated risk of excessive bleeding^[130]^.

These findings indicate that vitamin E’s antioxidant properties might support cognitive improvement and remyelination, particularly in conditions involving OS and neurodegeneration, warranting further research in PwMS.

## Coenzyme Q10 (CoQ10)

CoQ10, also called ubiquinone, is essential for mitochondrial energy production and stability, with notable neuroprotective properties that modulate OS and inflammation^[131–136]^. In animal models, CoQ10 supplementation has reduced age-related memory impairment, correlating with increased ATP levels, reduced ROS and lipid peroxidation, enhanced activity of antioxidative enzymes, and regulation of proteins involved in apoptosis and mitophagy in the hippocampus. suggesting potential cognitive benefits through mitochondrial support^[137]^.

In animal models of MS, CoQ10 has shown promising effects in reducing OS and inflammation. In a study on mice with EAE by Soleimani, *et al*, CoQ10 administration significantly reduced clinical symptoms of CNS neuroinflammation. Administration of CoQ10 lowered TNF-α levels, and shifted the Th1/Th2 interleukin balance toward an anti-inflammatory profile, highlighting CoQ10’s potential in suppressing inflammatory pathways involved in MS^[138]^. Similarly, Khalilian’s research on cuprizone-induced models of demyelination has shown that CoQ10 treatment can promote remyelination, increase the expression of myelin-associated proteins (MBP and Olig1), and reduce OS markers like TNF-α and IL-6^[139]^. Histological analyses confirmed enhanced remyelination, with improvements in TAC and SOD activity. Together, these studies suggest that by alleviating OS and inflammation, CoQ10 may offer neuroprotective and remyelinating benefits in the treatment of MS.

No clinical studies to date have specifically investigated CoQ10’s impact on cognitive functions in MS. However, several studies have supplemented PwMS with CoQ10 and investigated its effects on other symptoms of MS or biomarkers of disease activity. A study by Sanoobar et al. demonstrated that 12 weeks of CoQ10 supplementation (500 mg/day) significantly reduced key inflammatory markers, including TNF-α, IL-6, and MMP-9, in patients with RRMS, although it did not affect anti-inflammatory markers IL-4 and TGF-β^[140]^. This finding highlights CoQ10’s potential in targeting inflammation associated with MS. Additionally, clinical studies have reported that CoQ10 supplementation can alleviate fatigue and depression in people with MS, improvements that were linked to reductions in OS and inflammation^[141–143]^. Given the interconnected nature of depression, fatigue, and cognition in MS^[144–148]^, it is plausible that CoQ10 could also benefit cognitive health. This underscores the need for future research in this area.

## Discussion

**Figure 1.**
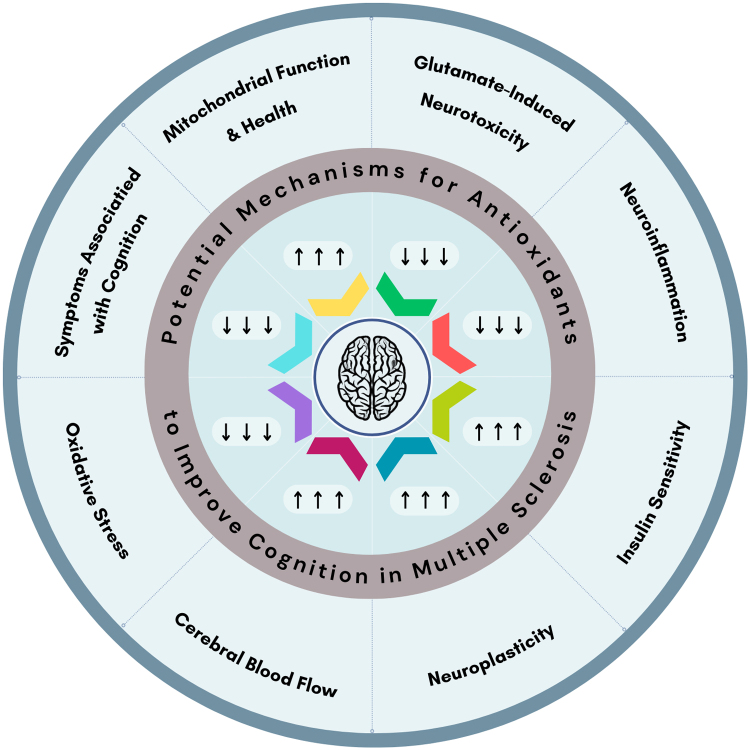
Potential underlying mechanisms mentioned in this review, by which antioxidants may improve cognition in patients with multiple sclerosis.

Based on the clinical literature review, evidence supporting the benefit of antioxidative supplementation for cognition in PwMS is controversial. Clinical studies investigating this matter were discussed for each antioxidant, and a summary is provided in Table 1. The underlying mechanisms of the observed effects were complex, encompassing pathways other than the antioxidative system. Furthermore, several of the antioxidants discussed were natural plant extracts (Gb and saffron) that comprise various compounds. This further complicates the understanding of the molecular mechanisms involved in the observed effects in clinical trials. In summary, the major mechanisms mentioned by which antioxidants may exert an effect on cognition were: (1) slowing disease progression by controlling OS in the CNS (by both direct and indirect antioxidative properties), (2) slowing disease progression by controlling neuroinflammation, (3) supporting mitochondrial function, (4) regulating neurotransmitters, especially glutamate, (5) enhancing CBF, (6) enhancing insulin sensitivity, (7) improving neuroplasticity, and (8) improving other symptoms that can negatively impact cognition (Fig. 1).

**Table 1:** Summary of clinical studies on cognitive effects of supplementing with antioxidants in patients with MS

Nutrient	Study authors (year)	Study design	Study population	Dose and duration	Cognitive outcomes
Melatonin	Jallouli, *et al* (2022)^a^	Double-blind, placebo-controlled, cross-over, randomized pilot trial	14 RRMS	6 mg PO, single dose	Improved MoCA compared with placebo (*P* = 0.008) in the following morning.
					No Improvements in SRT scores compared with placebo (*P* = 0.89).
	Roostaei, *et al* (2015)^b^	Double-blind, placebo-controlled, RCT	25 RRMS (13 melatonin, 12 placebo)	3 mg/day PO, 1y	Improved cognitive subdomain of MFIS (*P* = 0.006).
					No improvement in PASAT-2 (*P* = 0.81) and PASAT-3 (*P* = 0.33).
*Ginkgo biloba*	Noroozian, *et al* (2011)^c^	Open trial	30 PwMS (30 intervention, no placebo group)	240 mg/day PO, 8 w	Improved Wechsler memory scale (*P* = 0.001).
	Lovera, *et al* (2007)^d^	Double-blind, placebo-controlled, randomized pilot trial	39 PwMS (20 intervention, 19 placebo)	240 mg/day PO, 12 w	Improved in SCWT compared with placebo (*P* = 0.015).
					No improvement in CVLT-II long delay free recall, COWAT, PASAT, SDMT, and UFOV compared with placebo (*P* ≥ 0.05).
					Improved scores in the Retrospective Memory Scale of the PDQ (*P* = 0.015).
	Lovera, *et al* (2012)^e^	Double-blind, placebo-controlled, RCT	120 PwMS (61 intervention, 59 placebo)	240 mg/day PO, 12 w	No improvements in SCWT, CVLT-II long delay free recall, COWAT, and PASAT-2 compared with placebo (*P* ≥ 0.05).
					No improvements in PDQ, MSNSQ, and MFIS compared with placebo (*P* ≥ 0.05).
Omega-3 fatty acids	Jelinek, *et al* (2019)^f^	Cross-sectional analysis of an international cohort	2464 PwMS	History of taking supplements in the past 12 m	Patients taking whether flaxseed or fish oil had better scores in a subscale of the MSQOL-54 questionnaire, while controlled for age, sex, disability, a recent relapse, fatigue, depression, and education (*P* = 0.022). The subscale contains questions about memory and concentration
	Nag, *et al* (2021)^g^	Cross-sectional	1108 PwMS	History of taking supplements in the past 6 m	Neither progressive nor RRMS patients taking omega-3 supplements did not score better on self-reported cognitive outcomes, as measured by Neuro-QoL, while controlled for age, sex, disease duration, disability, education, and BMI (*P* ≥ 0.05 for both).
	Saxby, *et al* (2023)^h^	Single-blind, RCT	77 RRMS (39 Wahls diet and 38 Swank diet)	cod liver oil 5 g/day PO in both groups, 24 w	Both groups improved in 12 and 24 weeks PDQ (*P* ≤ 0.0002 for both groups in both times) and 24 weeks SDMT (*P* < 0.05 for both groups). The increase was not significantly different between the two groups (*P* > 0.05).
					The 12 week changes of serum EPA + DHA and serum EPA were associated with changes in SDMT (*P* = 0.04 and 0.02, respectively). The serum fatty acid changes had no mediation effect on the effect of the diets on SDMT or PDQ (*P* > 0.05).
Lipoic acid	Spain, *et al* (2017)^i^	Triple-blind, placebo-controlled, randomized pilot trial	50 SPMS (26 lipoic acid, 24 placebo)	1200 mg/day PO,96 w	68% reduction in annualized percent change brain volume (*P* = 0.002),
					SDMT scores did not improve (*P* = 0.59).
N-Acetylcysteine	Shahrampour, *et al* (2021)^j^	Open, controlled, randomized pilot trial	24 PwMS (12 intervention, 12 standard of care)	50 mg/kg IV once per week, 1 g PO in the other 6 days, 2 m	Improved MHI and PDQ compared with control group (*P* < 0.05 for both).
Lutein	Martell, *et al* (2023)^k^	Single-blind, placebo-controlled, RCT	21 RRMS (12 lutein, 9 placebo)	20 mg/day, 4 m	No improvement in Eriksen flanker task + EEG, SDMT, and spatial reconstruction task compared with placebo (*P* > 0.05).
					MPOD change in the treatment group was correlated with change in incongruent Eriksen flanker task accuracy (*P* = 0.03) and change in object-location binding in the spatial reconstruction task (*P* = 0.02).
Vitamin A	Bitarafan, *et al* (2016)^l^	Double-blind, placebo-controlled, RCT	93 RRMS (47 retinyl palmitate, 46 placebo)	25000IU/day for 6 m, then 10000IU/day for another 6 m	Improvement in the cognitive subdomain of MFIS compared with placebo (*P* = 0.02).
	Bitarafan, *et al* (2015)^m^	Double-blind, placebo-controlled, RCT	93 RRMS (47 retinyl palmitate, 46 placebo)	25000IU/day for 6 m, then 10000IU/day for another 6 m	Improved PASAT compared with placebo (*P* = 0.03).
	Ruschil, *et al* n	Open, controlled, RCT	53 progressive MS (3 intervention and 50 control)	High-dose all-trans retinoic acid for a minimum of 300 days	No significant improvement in PASAT (*P* > 0.05).
Saffron	Doosti, *et al* (2019)^o^	Triple-blind, placebo controlled, RCT	50 RRMS	1 year	Improved COWAT (*P* = 0.006) and NAART^a^ (*P* = 0.015) compared with placebo.
					No significant between group improvement in other components of MACFIMS (*P* > 0.05).
Crocin + Selenium nanoparticles	Rezaeimanesh, *et al* (2023)^p^	Triple-blind, placebo controlled, RCT	60 PwMS (30 crocin + selenium, 30 placebo)	5.74 mg crocin + 55mcg selenium, 12 w	No significant between-group improvement in BICAMS (CVLT-II, BVMT, SDMT) (*P* > 0.05)

MS: Multiple sclerosis; RRMS: Relapsing-remitting MS; PO: per os, by mouth, taken orally; RCT: Randomized clinical trial; y: year/years; PwMS: Patients with MS; w: week/weeks; m: month/months; EPA: Eicosapentaenoic acid; DHA: Docosahexaenoic acid; EEG: Electroencephalography; MPOD: Macular pigment optical density.

Abbreviations for cognitive measures: Cognitive Measures: MoCA: Montreal Cognitive Assessment; SRT: Simple reaction time; MFIS: Modified fatigue impact scale; PASAT: Paced auditory serial addition test; SCWT: Stroop color and word test; CVLT-II: California verbal learning test, second edition; COWAT: Controlled oral word association test; SDMT: Symbol digit modalities test; UFOV: Useful field of view; PDQ: Perceived deficits questionnaire; MSNSQ: Multiple sclerosis neuropsychological screening questionnaire; MSQOL: Multiple sclerosis quality of life; MHI: Mental health inventory; NAART: North American adult reading test; MACFIMS: Minimal assessment of cognitive function in multiple sclerosis; BICAMS: Brief International Cognitive Assessment for MS.

^a^ Jallouli S, Ghroubi S, Dhia IB, Yahia A, Elleuch MH, Sakka S, et al. Effect of melatonin intake on postural balance, functional mobility and fall risk in persons with multiple sclerosis: a pilot study. Int J Neurosci. 2022:1-11.

^b^ Roostaei T, Sahraian MA, Hajeaghaee S, Gholipour T, Togha M, Siroos B, et al. Impact of Melatonin on Motor, Cognitive and Neuroimaging Indices in Patients with Multiple Sclerosis. Iran J Allergy Asthma Immunol. 2015;14(6):589-95.

^c^ Noroozian M, Mohebbi-Rasa S, Tasviechi AK, Sahraian M, Karamghadiri N, Akhondzadeh S. Ginkgo biloba for Improvement of Memory and Quality of Life in Multiple Sclerosis: an Open Trial. Journal of Medicinal Plants. 2011;10:33-42.

^d^ Lovera J, Bagert B, Smoot K, Morris CD, Frank R, Bogardus K, et al. Ginkgo biloba for the improvement of cognitive performance in multiple sclerosis: a randomized, placebo-controlled trial. Mult Scler. 2007;13(3):376-85.

^e^ Lovera JF, Kim E, Heriza E, Fitzpatrick M, Hunziker J, Turner AP, et al. Ginkgo biloba does not improve cognitive function in MS: a randomized placebo-controlled trial. Neurology. 2012;79(12):1278-84.

^f^ Jelinek PL, Simpson S, Jr., Brown CR, Jelinek GA, Marck CH, De Livera AM, et al. Self-reported cognitive function in a large international cohort of people with multiple sclerosis: associations with lifestyle and other factors. Eur J Neurol. 2019;26(1):142-54.

^g^ Nag N, Yu M, Jelinek GA, Simpson-Yap S, Neate SL, Schmidt HK. Associations between Lifestyle Behaviors and Quality of Life Differ Based on Multiple Sclerosis Phenotype. J Pers Med. 2021;11(11).

^h^ Saxby SM, Haas C, Shemirani F, Titcomb TJ, Eyck PT, Rubenstein LM, et al. Association between improved serum fatty acid profiles and cognitive function during a dietary intervention trial in relapsing-remitting multiple sclerosis. International journal of MS care. 2023.

^i^ Spain R, Powers K, Murchison C, Heriza E, Winges K, Yadav V, et al. Lipoic acid in secondary progressive MS: A randomized controlled pilot trial. Neurol Neuroimmunol Neuroinflamm. 2017;4(5):e374.

^j^ Shahrampour S, Heholt J, Wang A, Vedaei F, Mohamed FB, Alizadeh M, et al. N-acetyl cysteine administration affects cerebral blood flow as measured by arterial spin labeling MRI in patients with multiple sclerosis. Heliyon. 2021;7(7):e07615.

^k^ Martell SG, Kim J, Cannavale CN, Mehta TD, Erdman JW, Jr., Adamson B, et al. Randomized, Placebo-Controlled, Single-Blind Study of Lutein Supplementation on Carotenoid Status and Cognition in Persons with Multiple Sclerosis. J Nutr. 2023;153(8):2298-311.

^l^ Bitarafan S, Saboor-Yaraghi A, Sahraian MA, Soltani D, Nafissi S, Togha M, et al. Effect of Vitamin A supplementation on fatigue and depression in multiple sclerosis patients: A double-blind placebo-controlled clinical trial. Iran J Allergy Asthma Immunol. 2016;15(1):13-9.

^m^ Bitarafan S, Saboor-Yaraghi A, Sahraian MA, Nafissi S, Togha M, Beladi Moghadam N, et al. Impact of Vitamin A Supplementation on Disease Progression in Patients with Multiple Sclerosis. Arch Iran Med. 2015;18(7):435-40.

^n^ Ruschil C, Dubois E, Stefanou M-I, Kowarik MC, Ziemann U, Schittenhelm M, et al. Treatment of progressive multiple sclerosis with high-dose all-trans retinoic acid–no clear evidence of positive disease modifying effects. Neurological Research and Practice. 2021;3:1-10.

^o^ Doosti R, Ghasemi-Sakha F, Saeedi R, Almasi-Hashiani A, Moghadasi AN, Sahraian MA, et al. A triple-blind, randomized controlled trial of Saffron in cognitive function of multiple sclerosis patients. Multiple Sclerosis Journal. 2019;25:884-.

^p^ Rezaeimanesh N, Rafiee P, Saeedi R, Khosravian P, Sahraian MA, Eskandarieh S, et al. The effect of crocin-selenium nanoparticles on the cognition and oxidative stress markers of multiple sclerosis patients: a randomized triple-blinded placebo-controlled clinical trial. Biometals. 2023.

Cellular defense mechanisms against OS in the CNS, especially the glutathione system, are abrupt in NDDs^[149,150]^. Therefore, protecting against OS in brain regions associated with cognition is likely to result in slowing cognitive decline in PwMS^[151]^. It is established that mitochondrial dysfunction is a key factor in the pathophysiology of axonal injury in MS. Demyelinated axons require higher amounts of ATP due to hyperexcitation, leading to a “virtual hypoxia.” Furthermore, neuroinflammation and the subsequent OS cause mitochondrial damage, aggravating the energy imbalance^[152–154]^. Antioxidants may control this cycle, guarding against free radicals and preserving mitochondrial function. Furthermore, some of the antioxidants reviewed here are directly involved in mitochondrial functions, such as DHA, LA, and CoQ10^[50,51,71,131]^. Besides virtual hypoxia, real hypoxia due to impaired CBF also leads to neuronal damage in MS. It was previously discussed that NAC and Gb may enhance the CBF, potentially protecting neurons from hypoxia^[20,88]^.

Moreover, several antioxidants, such as CoQ10, NAC, melatonin, Gb, and omega-3 fatty acids, can reduce neuroinflammation by suppressing the production of inflammatory cytokines^[13,20,55,80,136]^. Pro-inflammatory cytokines, which are known to be a crucial part of MS pathogenesis^[155]^, could lead to cognitive dysfunction by interacting with glial cells and inducing neurodegeneration^[177-180]^. Gb and LA have a regulatory function on NF-kB, which is responsible for pro-inflammatory cytokine release. In addition to the primary therapies, these antioxidants may be good choices as adjuvant agents to prevent neuroinflammation.

Besides the direct effects on cognition, some antioxidants also modulate other health factors that influence cognitive performance, such as fatigue, depression, and sleep quality. A large number of studies indicate that these factors adversely affect cognition^[156–158]^. One example is the effect of CoQ10 supplementation on alleviating fatigue and depression. Additionally, melatonin is used predominantly for improving sleep quality. As discussed, a single dose of melatonin can enhance cognition in the following morning, reflecting its instant effect by improving sleep quality^[16]^. Sleep deprivation itself is also associated with OS and inflammation, which can potentially account for significant differences over the long term^[159]^.

No cognitive benefits were observed in several of mentioned trials in this study. This can partly be due to the short follow-up duration of these studies. However, as discussed before, these antioxidants work through several different pathways in human body. Considering multiple pathological pathways associated with demyelination and neurodegeneration in MS, a combination of these compounds is more likely to have a significant benefit^[2]^. There are also related studies investigating antioxidant combination in multiple sclerosis. Few studies are done in this manner, which is expected as it would be harder to interpret the findings. In this review, we mentioned the study by Rezaeimanesh, *et al*, in which PwMS were supplemented with selenium-crocin nanoparticles^[160]^. Although this study showed no cognitive benefits, the duration of follow-up was three months, which is probably not enough for observing cognitive changes due to slowed disease progression. Therefore, we recommend adopting a therapeutic regimen that incorporates a comprehensive antioxidant formula encompassing several of the mechanisms discussed.

In summary, the current review highlights the knowledge gap regarding the cognitive effects of antioxidative treatments in PwMS. While OS is a major contributing factor to MS pathology, designing studies to establish a positive effect on cognition associated with antioxidant supplements presents several challenges. These challenges primarily stem from the long follow-up durations necessary to identify any effects, the multifaceted pathology of MS, and the complex mechanisms of action associated with each antioxidant, making it difficult to interpret the findings. However, the preclinical evidence is promising in many cases, indicating potential for further clinical research in this area.

## Data Availability

Data sharing is not applicable to this article, as it is a narrative review and does not involve original datasets.
